# Uptake and determinants of childhood vaccination status among children aged 0–12 months in three West African countries

**DOI:** 10.1186/s12889-023-15863-w

**Published:** 2023-06-06

**Authors:** Amadou Barrow, Ayobami Oyekunle Afape, Dalanda Cham, Precious Chidozie Azubuike

**Affiliations:** 1 Department of Public & Environmental Health, School of Medicine & Allied Health Sciences, University of The Gambia, Kanifing, The Gambia; 2 Department of Epidemiology, University of Florida, Gainesville, United States of America; 3 Epidemiology & Disease Control Unit, Ministry of Health, Kotu, The Gambia; 4 Kaduna State Primary Health Care and Development Board, Kaduna State, Nigeria; 5 Department of Public Health, University of Calabar, Calabar, Nigeria

**Keywords:** Children under 12 months, Vaccination, Uptake, Determinants, Childhood

## Abstract

**Background:**

Vaccination has long been recognized as one of the most effective ways to reduce child mortality. It has played a significant role, particularly for children, and is considered a major achievement and relevant in preventing childhood diseases worldwide. This study looks at the uptake and determinants of childhood vaccination status among children under the age of one year, for Gambia, Sierra Leon, and Liberia.

**Method:**

Data from 2019 to 20 Demographic and Health Survey (DHS) data from Gambia, Sierra Leone, and Liberia were pooled for the analysis used in this study. Data were obtained from a total weighted sample of 5,368 children aged 0–12 months through a stratified two-stage cluster sampling approach. A multivariable logistic regression model was used to assess the predictors of childhood vaccination uptake at 95% confidence interval (CIs) with computed adjusted odds ratios (aORs).

**Results:**

The weighted sample pooled prevalence of full vaccination among children under 12 months of age was 15.1% for males and 15.0% for females. After controlling for confounders in the regression model, factors that were found to be associated with vaccination status include children whose mothers attended postnatal care (PNC) visits had higher odds of being fully vaccinated (aOR = 1.23, 95% CI = 1.03–1.46), while children whose fathers had primary education (aOR = 0.67, 95% CI = 0.48–0.96), children whose households never watched TV (aOR = 0.68, 95% CI = 0.56–0.82) and children whose mothers attended 1–3 antenatal care (ANC) visits (aOR = 0.59, 95% CI = 0.45–0.79) had lower odds of being fully vaccinated.

**Conclusion:**

Childhood vaccination uptake was low among children under 12 months of age in these countries. Hence, there is a need to promote the uptake of vaccination across these three West African countries especially among rural dwellers.

## Introduction

Vaccination has long been recognized as one of the most effective ways to reduce child mortality [1]. It has played a significant role, particularly for children, and is considered a major achievement and relevant in preventing childhood diseases worldwide. In practice, the terms “vaccination” and “immunization” are interchangeable [2]. Improving access to immunization is critical to meeting the Sustainable Development Goals (SDGs). Childhood vaccination is highly effective in preventing vaccine-preventable diseases [1]. The discovery and introduction of the smallpox vaccine have greatly reduced disease prevalence worldwide, with 29 vaccine-preventable illnesses recommended by the World Health Organization (WHO) [3]. Despite the numerous ways to reduce the number of children dying from vaccine-preventable diseases, immunizations have improved immunity in 9 out of every 10 children [2]. Vaccines are very effective when major risk factors like malnutrition, air pollution and diarrheal diseases are eliminated, as well as treatment accessibility is improved.

Poor adherence to vaccination still remains a challenge in many regions across the world [4]. Globally, it is estimated that about 22.4 million children under one year of age were not vaccinated with the third dose of pentavalent vaccine (Diphtheria Pertussis-Tetanus-Hepatitis B-Haemophilus type b influenza vaccine (DPT-HB-Hib) [3]. One out of five African children is estimated to not receive all the necessary and basic vaccinations. Consequently, over 30 million African children under five still suffer from vaccine-preventable diseases (VPDs) every year. Approximately 58% of all deaths due to VPDs occur in children over half a million a year [5]. Several factors contribute to vaccines’ low coverage as highlighted in the literature. Households, lack of communication, and knowledge about immunization have been reported to be major contributors [6]. One of the major contributors to vaccine utilization in Africa highlighted is economic conditions, which affect the ability to afford and keep immunization record documents in good condition [7]. A study published in the literature looked into cultural perspectives as a contributor to low vaccination coverage. It was discovered to be linked with people’s misinformed perceptions of vaccines due to their cultural background [8]. In Pakistan, is linked to the father’s job as manual labour [9]. In addition, mothers’ perception of the severity of vaccine-preventable disease, as well as mothers’ beliefs do not influence the uptake as reported in a study conducted in Tanzania [10].

Sub-Saharan Africa is still dealing with a variety of program and policy challenges related to childhood immunization [11]. Numerous studies have been conducted to investigate factors associated with childhood immunization in Sub-Saharan Africa, some of which indicate parental attitudes, a lack of knowledge among health workers, and barriers that can be overcome by improving outreach services [12]. Furthermore, a study conducted in Burkina Faso discovered that education, both at the individual and community levels, is no longer associated with complete vaccination of children [13]. This demonstrates that there are differences in factors within the African region, such as the need for uptake. It has been observed that many countries in the sub-Saharan African region have proven to have a high vaccination coverage, but due to various limitations have not achieved this upscaled coverage, necessitating the need to study underlying factors [11].

Several West African immunization programs have made significant progress in increasing vaccination coverage, which is expected to have a significant impact on the reduction of vaccine-preventable diseases [1]. The Gambia, Sierra Leon, and Liberia for example, have shown that the immunization strategies are effective when there are good political and cultural will; however, vaccination programs should be constantly monitored and evaluated by the Ministries of Health. To raise societal awareness and vaccine acceptance, strong community-based health education efforts are desperately needed as part of initiatives to increase vaccine service utilization for these high-risk classes. This study examines the uptake and determinants of childhood vaccination status among children under the age of one, using the available DHS data for Gambia, Sierra Leon, and Liberia.

## Methods

### Data source

The 2019-20 Demographic and Health Survey (DHS) data from Gambia, Sierra Leone, and Liberia were pooled for the analysis. In the three countries, the DHS used a stratified two-stage cluster sampling approach to create a population-based sample. Following the probability proportional to the size of the Enumerated Areas (EAs), 281 (The Gambia), 325 (Liberia), and 578 (Sierra Leone) clusters/EAs were selected in the first stage of the survey. The second stage involved a methodical selection of 25 (The Gambia), 30 (Liberia), 24 (Sierra Leone) households from each cluster/EA and only 8,362 (The Gambia), 5, 704 (Liberia), 9,889 (Sierra Leone) women of reproductive age with children less than 60 months were interviewed successfully. In DHS, specific questions were asked to women about children’s health. Questions related to immunization coverage are of particular interest. We pooled the DHS survey data of the three countries, and a total weighted sample of 5,368 children aged 0–12 months was included in the study. In the three countries, through the USAID-funded MEASURE DHS programme, ICF International provided technical and financial assistance to the Ministry of Health in collaboration with the Bureau of Statistics of each country that implemented the survey.

### Variable selection and measurement

***Outcome variables***. The study outcome variable was childhood vaccination status among children aged 0–12 months. This was classified into two categories: “Fully vaccinated” a child under 23 months who received WHO recommended vaccination against tuberculosis (also known as BCG), three doses of DPT-HepB-Hib (Penta), three doses of polio vaccines, and one dose of vaccination against measles; “Partially vaccinated” who missed at least one of any of the doses of the routine vaccines before turning 1 year or within 12–23 months old [14–18].

***Explanatory variables***. Thirteen independent variables were utilized in the study based on a thorough literature review and datasets availability [14–16]; the variables are listed in Table 1.

The WHO framework on epidemiology of the unimmunized child [19, 20] describes the different factors affecting child’s immunization into four main categories: health care immunization system, communication and information, family characteristics, and parental attitudes and knowledge [21]. In our study, the immunization system category included the distance to health facility, and the need to take transportation. The communication and information category included: use of mass media according to the levels of access and source (radio, TV and newspapers), family characteristics included the followings variables: mother’s and father’s education level, mother’s age at childbirth, marital status, household level of poverty assessed by the wealth quintile, ethnic group, religion, child gender, birth order, urban/rural residence (urban/rural), and region of residence. Variables on familiarity and use of other health care services such as antenatal care during pregnancy, postnatal care, and the relative distance to the closest health center represented the parental attitudes/knowledge. Finally, we included the gender relationship such as the involvement of women in household decision making.

**Table 1:** Definition of independent variables used in the analysis

Variable	Definition and coding
Country	1 = Gambia; 2 = Sierra Leone; 3 = Liberia
Sex of child	1 = Male; 2 = Female
Number of under 5 children	1 = 0–1; 2 = 2–3; 3 = ≥ 4
Age of mother	1 = 15–24; 2 = 25–34; 3 = > 35
Educational level of mother	0 = No Education; 1 = Primary; 2 = Secondary and above
Age of father	1 = 15–24; 2 = 25–34; 3 = > 35
Educational level of father	0 = No Education; 1 = Primary; 2 = Secondary and above
Marital Status	0 = Not currently married; 1 = Currently married/living with partner; 2 = Widowed/Divorced
Wealth index	1 = Poorest; 2 = Poorer; 3 = Middle; 4 = Richer; 5 = Richest
Mother’s occupation	0 = Working; 1 = No working
Father’s occupation	0 = Working; 1 = No working
Media exposure	0 = Never; 1 = Sometimes
ANC visit	0 = No; 1 = 1–3; 2 = ≥ 4
PNC Visit	0 = No; 1 = Yes
Parity	1 = 1–3; 2 = 4–6; 3 = ≥ 7
Place of delivery	1 = at hospital; 2 = at home
Distance to health facility	1 = No problem; 2 = Big problem
Religion	1 = Islam; 2 = Christianity; 3 = Others
Place of residence	1 = Urban; 2 = Rural
Decision making on vaccination	1 = Mother; 2 = Father; 3 = Both

### Statistical analysis

Stata survey (‘svy’) module was used to adjust for stratification, clustering and sampling weights provided in the dataset. The data was weighted (v005/1,000,000) throughout the analysis to ensure DHS sample representativeness and to obtain reliable estimates and standard errors before data analysis. Descriptive statistics were used to describe the level of immunization coverage by socio-demographic characteristics, the distributions were expressed as frequencies and percentages. Bivariate and multivariable logistic regression analyses were conducted to identify the determinants of full immunization. Bivariate analyses were used to examine the crude association between each independent variable and full vaccination. Multivariable logistic regression was used to examine the adjusted association between each independent variable and full childhood vaccination. Binary logistic regression was chosen because our dependent variable was dichotomous (i.e., 0 - partially vaccinated and 1- fully vaccinated). Variables in bivariate analysis with p-values less than 0.05 were entered into the multivariable analysis. Adjusted odds ratio (aOR) and 95% confidence Interval (CI) were used to assess the strength of associations between the outcome and the independent variables. The threshold for statistical significance was set at p < 0.05. We used the Bayesian Deviance Information Criterion (BIC) to assess the goodness of fit of the model. Variance Inflation Factor (VIF) was applied to test for multicollinearity. All the study data were analyzed using Stata version 17 and IBM SPSS version 25.

### Ethical approval

The datasets used in this research were population-based datasets that are freely available in the public domain. For reasons of confidentiality, specific characteristics that could be used to identify participants in the study were excluded. As a secondary study, MEASURE DHS/ICF International granted the authors permission to use the datasets.

## Results

### Socio-demographic characteristics of infants’ mothers in Gambia, Liberia and Sierra Leone

Based on the result from the pooled data as shown in Table 2, a total of 5,368 children aged 0 and 12 months were pooled across the three West African countries for the study. Child characteristics showed that about half (52.1%) of the children in the study sample were males and 47.9% were females. The family/parental characteristics showed that the mean age of child’s mother and father (± SD) was 27 years (± 6.8) and 38 years (± 11.1) respectively, almost half of the mothers (44.7%) and 49.5% of the fathers had no formal education. 86.3% of the mothers had more than 4 ANC visit, 42.8% never attended PNC, 79.0% delivered in public hospitals and 53.8% of the fathers made decision on vaccination. Household characteristics showed that among the respondents, 50.3% lived in rural area, 22.9% were among the poorest wealth quintile and 61.5% practiced Islam. Communication and information characteristics showed that 92.9% never read newspaper, 38.7% sometimes listened to radio, and 42.9% sometimes watched TV.

**Table 2:** Characteristics of weighted sample population

Variables	Pooled Data		Gambia		Liberia		Sierra Leone	
	n	%	n	%	n	%	n	%
**Child Sex**								
Male	2794	52.1	975	52.8	634	51.4	1185	51.9
Female	2573	47.9	873	47.2	600	48.6	1100	48.1
**No of under 5 children**								
0–1	1708	31.8	307	16.6	564	45.7	837	36.6
2–3	2690	50.1	818	44.3	600	48.6	1272	57.7
≥ 4	970	18.1	723	39.1	71	5.7	176	7.7
**Mother’s age**								
< 24	1956	36.4	564	30.5	533	43.2	859	37.6
25–34	2379	44.3	919	49.7	491	39.8	969	42.4
≥ 35	1034	19.3	365	19.8	211	17.1	458	20.0
**Mother’s education**								
No education	2398	44.7	824	44.6	404	32.7	1170	51.2
Primary	972	18.1	332	18.0	306	24.8	334	14.6
Secondary	1819	33.9	623	33.7	470	38.1	726	31.8
Tertiary	178	3.3	69	3.7	54	4.4	55	2.4
**Mother’s currently working**								
No	2233	41.6	984	53.2	554	44.9	695	30.4
Yes	3136	58.4	864	46.8	681	55.1	1591	69.6
**Husband/Partner’s age**								
< 24	228	5.1	28	1.6	80	9.9	120	6.3
25–34	1424	31.9	463	26.5	329	40.6	632	33.0
≥ 35	2818	63.0	1255	71.9	401	49.5	1162	60.7
*Mean (± SD)*	38.4 (*±* 11.2)		40.6 (*±* 11.2)		34.8 (*±* 8.7)		37.9 (*±* 11.7)	
**Husband/Partner’s education**								
No education	2081	49.5	851	53.5	214	28.1	1016	54.9
Primary	363	8.6	104	6.5	119	15.6	140	7.6
Secondary	1400	33.3	492	30.9	363	47.5	546	29.5
Tertiary	357	8.5	143	9.0				
**Residence**								
Urban	2668	49.7	1169	63.3	687	55.7	812	35.5
Rural	2699	50.3	679	36.7	547	44.3	1473	64.5
**Marital status**								
Single	761	14.2	83	4.5	361	29.2	317	13.9
Married	4470	83.3	1746	94.5	810	65.6	1914	83.8
Widowed/Divorced	137	2.5	19	1.0	64	5.2	54	2.4
**Wealth**								
Poorest	1229	22.9	430	23.3	282	22.9	517	22.6
Poorer	1189	22.2	407	22.0	266	21.6	516	22.6
Middle	1043	19.4	375	20.3	225	18.2	443	19.4
Richer	1002	18.7	316	17.1	239	19.4	447	19.6
Richest	904	16.8	319	17.3	222	18.0	363	15.9
**Religion**								
Islam	3293	61.5	1812	98.1	1031	84.2	450	19.7
Christianity	2051	38.3	35	1.9	181	14.8	1835	80.3
Other	14	0.3	0	0.0	13	1.1	1	0.0
**Watch TV**								
Sometimes	2305	42.9	1400	75.8	394	31.9	511	22.4
Never	3064	57.1	448	24.2	841	68.1	1775	57.1
**Listen to Radio**								
Sometimes	2978	38.7	1417	76.7	676	54.8	885	38.7
Never	2388	61.3	430	23.3	558	45.2	1400	61.3
**Read Newspaper**								
Sometimes	383	7.1	142	7.7	117	9.5	124	5.4
Never	4985	92.9	1706	92.3	1117	90.5	2162	94.6
**ANC visit**								
No	51	1.0	8	0.4	13	1.1	30	1.3
1–3	666	12.7	363	20.0	123	10.1	180	8.1
4>	4534	86.3	1443	79.5	1078	88.8	2013	90.6
**PNC Visit**								
No	2995	57.2	818	45.1	958	78.9	1219	54.9
Yes	2244	42.8	992	54.7	255	21.0	997	44.9
**Parity**								
1–3	3183	59.3	1010	54.7	780	63.2	1393	60.9
4–6	1645	30.6	586	31.7	356	28.8	703	30.8
7>	540	10.1	252	13.6	98	7.9	190	8.3
**Place of delivery**								
Home	720	13.4	202	10.9	180	14.6	338	14.8
Public hospital	4241	79.0	1479	80.1	865	70.0	1897	83.0
Private hospital	407	7.6	166	9.0	190	15.4	51	2.2
**Distance to health facility**								
Big problem	2054	38.3	532	28.8	402	32.6	1120	49.0
Not a big problem	3313	61.7	1315	71.2	832	67.4	1166	51.0
**Decision making on vaccination**								
Mother	499	11.2	218	12.5	142	17.5	139	7.3
Father	2405	53.8	1018	58.3	212	26.2	1175	61.4
Both	1565	35.0	509	29.2	456	56.3	600	31.3

Table 3: **Bivariate analysis of factors associated with childhood vaccination among infant aged 0–12 months**.

Similar proportions of male (15.1%) and female children (15.0%) reportedly had full vaccination. Children whose mother were 25–34 years (16.3%) and 35 & above (14.4%) had higher proportion of full vaccination compared to those less than 24 years (13.9%) as shown in Table 3. Children whose mother had no education (14.4%) had lower full vaccination status compared to those with secondary (16.1%) and tertiary (16.1%) education. The proportion of full vaccination was higher among children who lived in urban area (15.8%) compared to those who resided in the rural area (14.3%). Higher proportion of full vaccination was recorded among children whose mothers were married (15.4%) compared to those single (13.8%) or widowed/divorced (12.3%). Higher proportion of full vaccination was recorded among those who sometimes watched TV (18.1%) or listened to radio (15.8%) or read newspaper (16.2%). The higher proportion of full vaccination was recorded among children whose mother had no ANC visit (19.6%) and had PNC visit (16.5%). Lower proportion of full vaccination status was recorded among those whose mothers delivered at home (12.8%) and had big problem about the distance to the health facility (12.8%). Bivariate analysis indicated that father’s education, religion, watching TV and listening to radio, ANC visit, PNC visit, distance to health facility were significant risk factors associated with full vaccination among children 0–12 months in three West African countries.

**Table 3:** Bivariate analysis of the association between the explanatory variables and childhood vaccination among children aged 0–12 months in three West African countries

	Pooled data			Gambia			Liberia			Sierra Leone		
	Partially vaccinated	Fully vaccinated		Partially vaccinated	Fully vaccinated		Partially vaccinated	Fully vaccinated		Partially vaccinated	Fully vaccinated	
**Child Sex**			0.861			0.480			0.265			0.168
Male	2372(84.9%)	423(15.1%)		778(79.8%)	197(20.2%)		550(86.8%)	84(13.2%)		1044(88.0%)	142(12.0%)	
Female	2188(85.0%)	385(15.0%)		708(81.1%)	165(18.9%)		533(88.8%)	67(11.2%)		947(86.1%)	153(13.9%)	
**No of under 5 children**			0.380			0.024*			0.107			0.275
0–1	1461(85.5%)	247(14.5%)		242(78.8%)	65(21.2%)		494(87.6%)	70(12.4%)		725(86.6%)	112(13.4%)	
2–3	2267(84.3%)	423(15.7%)		640(78.2%)	178(21.8%)		522(87.0%)	78(13.0%)		1105(86.9%)	167(13.1%)	
≥ 4	832(85.8%)	138(14.2%)		604(83.5%)	119(16.5%)		67(95.75)	3(4.3%)		161(91.0%)	16(9.0%)	
**Mother’s age**			0.072			0.390			0.099			0.353
< 24	1683(86.1%)	272(13.9%)		464(82.3%)	100(17.7%)		477(89.7%)	55(10.3%)		742(86.4%)	117(13.6%)	
25–34	1991(83.7%)	388(16.3%)		731(79.5%)	188(20.5%)		419(85.3%)	72(14.7%)		841(86.8%)	128(13.2%)	
≥ 35	885(85.6%)	149(14.4%)		290(79.5%)	75(20.5%)		187(88.6%)	24(11.4%)		408(89.1%)	50(10.9%)	
**Mother’s education**			0.417			0.946			0.003*			0.373
No education	2051(85.5%)	347(14.5)		658(79.9%)	166(20.1%)		363(90.1%)	40(9.9%)		1029(87.9%)	141(12.1%)	
Primary	833(85.6%)	140(14.4%)		271(81.4%)	62(18.6%)		280(91.5%)	26(8.5%)		282(84.4%)	52(15.6%)	
Secondary	1527(83.9%)	292(16.1%)		501(80.5%)	121(19.5%)		395(84.0%)	75(16.0%)		631(86.8%)	96(13.2%)	
Tertiary	148(83.1%)	30(16.9%)		56(80.0%)	14(20.0%)		43(81.1%)	10(18.9%)		49(89.1%)	6(10.9%)	
**Mother’s currently working**			0.966			0.073			0.575			0.828
No	1895(84.9%)	337(15.1%)		806(81.9%)	178(18.1%)		483(87.2%)	71(12.8%)		606(87.3%)	88(12.7%)	
Yes	1895(84.9%)	337(15.1%)		679(78.6%)	185(21.4%)		600(88.2%)	80(11.8%)		1384(87.0%)	207(13.0%)	
**Husband/Partner’s age**			0.251			0.518			0.383			0.478
< 24	194(85.1%)	34(14.9%)		20(71.4%)	8(28.6%)		73(91.3%)	7(8.7%)		101(84.2%)	19(15.8%)	
25–34	1224(85.9%)	201(14.1%)		372(80.3%)	91(19.7%)		294(89.4%)	35(10.6%)		558(88.2%)	75(11.8%)	
≥ 35	2366(84.0%)	452(16.0%)		1004(80.0%)	251(20.0%)		348(86.8%)	53(13.2%)		1014(87.3%)	452(16.0%)	
**Husband/Partner’s education**			0.023*			0.065			0.001*			0.450
No education	1771(85.1%)	310(14.9%)		684(80.4%)	167(19.6%)		196(91.6%)	18(8.4%)		891(87.7%)	125(12.3%)	
Primary	323(89.0%)	40(11.0%)		92(88.5%)	12(11.5%)		107(89.9%)	12(10.1%)		124(88.6%)	16(11.4%)	
Secondary	1179(84.2%)	221(15.8%)		391(79.5%)	101(20.5%)		319(88.9%)	43(11.9%)		469(85.9%)	77(14.1%)	
Tertiary	289(81.0%)	68(19.0%)		107(74.8%)	36(25.2%)		49(73.1%)	18(26.9%)		133(90.5%)	14(9.5%)	
**Residence**			0.105			0.141			0.005*			0.874
Urban	2246(84.2%)	423(15.8%)		952(81.4%)	217(18.6%)		588(85.5%)	100(14.5%)		706(86.9%)	106(13.1%)	
Rural	2314(85.7%)	385(14.3%)		533(78.6%)	145(21.4%)		496(90.7%)	51(9.3%)		1285(87.2%)	189(12.8%)	
**Marital status**			0.357			0.118			0.734			0.412
Single	655(86.2%)	105(13.8%)		73(88.0%)	10(12.0%)		313(86.9%)	47(13.1%)		269(84.9%)	48(15.1%)	
Married	3783(84.6%)	687(15.4%)		1395(79.9%)	351(20.1%)		715(88.3%)	95(11.7%)		1673(87.4%)	241(12.6%)	
Widowed/ Divorced	121(87.7%)	17(12.3%)		17(89.5%)	2(10.5%)		55(85.9%)	9(14.1%)		49(89.1%)	6(10.9%)	
**Wealth**			0.388			0.776			0.001*			0.343
Poorest	1055(85.8%)	174(12.9%)		341(79.3%)	89(20.7%)		263(93.3%)	19(6.7%)		451(87.2%)	66(12.8%)	
Poorer	1011(85.0%)	179(15.0%)		323(79.2%)	85(20.8%)		237(89.1%)	29(10.9%)		451(87.4%)	65(12.6%)	
Middle	896(85.9%)	147(14.1%)		301(80.3%)	74(19.7%)		201(89.3%)	24(10.7%)		394(88.9%)	49(11.1%)	
Richer	847(84.6%)	154(15.4%0		259(82.0%)	57(18.0%)		198(82.8%)	41(17.2%)		390(87.4%)	56(12.6%)	
Richest	750(83.1%)	153(16.9%)		261(82.1%)	57(17.9%)		184(82.9%)	38(17.1%)		305(84.0%)	58(16.0%)	
**Religion**			0.001*			0.358			0.024*			0.001*
Islam	2723(82.7%)	570(17.3%)		1459(80.5%)	353(19.5%)		895(86.8%)	136(13.2%)		369(82.0%)	81(18.0%)	
Christianity	1816(88.5%)	235(11.5%)		26(74.3%)	9(25.7%)		169(93.4%)	12(6.6%)		1621(88.3%)	214(11.7%)	
Other	14(93.3%)	1(6.7%)		1485(80.4%)	363(19.6%)		13(92.9%)	12(6.6%)		1(100.0%)	0(0.0%)	
**Watch TV**			0.001*			0.865			0.003*			0.024*
Sometimes	1886(81.9%)	418(18.1%)		1127(80.5%)	273(19.5%)		329(83.7%)	64(16.3%)		1561(87.9%)	214(12.1%)	
Never	2674(87.3%)	390(12.7%)		359(80.1%)	89(19.9%)		754(89.7%)	87(10.3%)		430(84.1%)	81(15.9%0	
**Listen to Radio**			0.088			0.226			0.058			0.427
Sometimes	2508(85.9%)	470(15.8%)		1148(81.0%)	269(19.0%)		583(86.2%)	93(13.8%)		777(87.8%)	108(12.2%)	
Never	2052(85.9%)	337(14.1%)		337(78.4%)	93(21.6%)		501(89.8%)	57(10.2%)		1214(86.7%)	108(13.3%)	
**Read Newspaper**			0.505			0.039*			0.010*			0.001*
Sometimes	320(83.8%)	62(16.2%)		104(73.8%)	37(26.2%)		94(80.3%)	23(19.7%)		122(98.4%)	2(1.6%)	
Never	4239(85.0%)	746(15.0%)		1381(80.9%)	325(19.1%)		989(88.5%)	128(11.5%)		1869(86.4%)	293(13.6%)	
**ANC visit**			0.004*			0.002*			0.004*			0.002*
No	41(80.4%)	10(19.6%)		8(100.0%)	0(0.0%)		13(100.0%)	0(0.0%)		20(66.7%)	10(33.3%)	
1–3	595(89.1%)	73(10.9%)		314(86.3%)	50(13.7%)		118(95.9%)	5(4.1%)		163(90.1%)	18(9.9%)	
4>	3820(84.3%)	713(15.7%)		1137(78.8%)	306(21.2%)		932(86.5%)	145(13.5%)		1751(87.0%)	262(13.0%)	
**PNC Visit**			0.035*			0.152			0.562			0.785
No	2576(85.8%)	428(14.2%)		674(81.9%)	149(18.1%)		837(83.3%)	122(12.7%)		1065(87.2%)	157(12.8%)	
Yes	1876(83.6%)	367(16.5%)		785(79.2%)	206(20.8%)		226(88.6%)	29(11.4%)		865(86.8%)	132(13.2%)	
**Parity**			0.683			0.882			0.161			0.401
1–3	2688(84.5%)	494(15.5%)		805(79.8%)	204(20.2%)		684(87.7%)	96(12.3%)		1199(86.1%)	194(13.9%)	
4–6	1412(85.9%)	231(14.1%)		478(81.6%)	108(18.4%)		314(88.5%)	41(11.5%)		620(88.3%)	82(11.7%)	
7>	458(84.85)	82(15.2%0		202(80.2%)	50(19.8%)		85(86.7%)	13(13.3%)		171(90.0%)	19(10.0%)	
**Place of delivery**			0.164			0.333			0.631			0.324
Home	627(87.2%)	92(12.8%)		165(81.7%)	37(18.3%)		161(89.9%)	18(10.1%)		301(89.1%)	37(10.9%)	
Public hospital	3584(84.5%)	657(15.5%)		1180(79.8%)	299(20.2%)		756(87.4%)	109(12.6%)		1648(86.9%)	249(13.1%)	
Private hospital	349(85.5%)	59(14.5%)		140(84.3%)	26(15.7%0		167(87.4%)	24(12.6%)		42(82.4%)	9(17.6%)	
**Distance to health facility**			0.014*			0.925			0.544			0.029*
Big problem	1777(86.5%)	278(13.5%)		427(80.3%)	105(19.7%)		357(88.6%)	46(11.4%)		993(88.7%)	127(11.3%)	
Not a big problem	2783(84.0%)	530(16.0%)		1058(80.5%)	257(19.5%)		727(87.4%)	105(12.6%)		998(85.6%)	168(14.4%)	
**Decision making on vaccination**			0.975			0.896			0.726			0.825
Mother	423(84.6%)	77(15.4%)		172(78.9%)	46(21.1%)		129(90.2%)	14(9.8%)		122(87.8%)	17(12.2%)	
Father	2033(84.5%)	372(15.5%)		817(80.3%)	201(19.75)		185(87.7%)	26(12.3%)		1031(87.7%)	145(12.3%)	
Both	1327(84.8%)	238(15.2%)		406(79.8%)	103(20.2%)		401(87.9%)	55(12.1%)		520(86.7%)	80(13.3%)	

*=Statistical significance p < 0.05

### Determinants of childhood vaccination among infant aged 0–12 months in the three west african countries

Table 4 shows the binary logistic regression results on the factors associated with childhood full vaccination in three West African countries. In the adjusted model, children whose mother attended PNC visit had higher odds (aOR = 1.23, 95% CI = 1.03–1.46) of being fully vaccinated compared to those who had no visit. Religion was significantly associated with full vaccination. Children whose father had primary education had lower odds (aOR = 0.67, 95% CI = 0.48–0.96) of being fully vaccinated compared to those who had no education. Children whose household never watched TV had lower odds (aOR = 0.68, 95% CI = 0.56–0.82) of full vaccination compared to those who watched sometimes. Finally, also children whose mother attended one to three ANC visit had lower odds (aOR = 0.59, 95% CI = 0.45–0.79) of being fully vaccinated compared to those who had more than four visit.

**Table 4:** Binary logistic regression analysis of factors associated with childhood vaccination among Children 0–12 months

	Pooled data	Gambia	Liberia	Sierra Leone
	Partially vs. Fully Vaccinated	Partially vs. Fully Vaccinated	Partially vs. Fully Vaccinated	Partially vs. Fully Vaccinated
	aOR (95%CI)	aOR (95%CI)	aOR (95%CI)	aOR (95%CI)
**PNC Visit**				
No *(Ref)*	1			
Yes	1.23(1.03–1.46)*			
**Distance to health facility**				
Not a big problem *(Ref)*	1			1
Big problem	0.97(0.81–1.17)			0.84(0.65–1.08)
**Religion**				
Islam *(Ref)*	1		1	1
Christianity	0.66(0.55–0.82)*		0.26(0.11–0.63)*	0.59(0.45–0.79)
Other	0.53(0.04–6.89)		0.74(0.54–10.1)	0.0(0.0-.)
**Watch TV**				
Sometimes *(Ref)*	1		1	1
Never	0.68(0.56–0.82)*		0.42(0.25–0.71)*	0.71(0.53–0.95)*
**Husband/Partner’s education**				
No education *(Ref)*	1		1	
Primary	0.67(0.48–0.96)*		1.06(0.46–2.46)	
Secondary	1.01(0.83–1.22)		0.78(0.38–1.59)	
Tertiary	1.12(0.87–1.58)		1.15(0.43–3.04)	
**ANC visits**				
No *(Ref)*	1.19(0.52–2.75)	0.0(0.0-.)	0.0(0.0-.)	2.79(1.25–6.23)*
1–3	0.59(0.45–0.79)*	0.59(0.43–0.82)*	0.34(0.11–1.03)	0.68(0.41–1.13)
4 & above	1	1	1	1
**Read Newspaper**				
Sometimes *(Ref)*		1	1	1
Never		0.72(0.48–1.07)	1.09(0.52–2.29)	12.1(2.94–49.95)*
**No of under 5 children**				
0–1 *(Ref)*		1		
2–3		1.03(0.74–1.42)		
≥ 4		0.79(0.57–1.12)		
**Mother’s education**				
No education *(Ref)*			1	
Primary			0.88(0.42–1.86)	
Secondary			1.25(0.61–2.55)	
Tertiary			1.31(0.39–4.31)	
**Residence**				
Urban *(Ref)*			1	
Rural			1.50(0.76–2.97)	
**Wealth**				
Poorest *(Ref)*			1	
Poorer			1.25(0.57–2.73)	
Middle			1.19(0.48–2.99)	
Richer			2.78(1.05–7.35)*	
Richest			3.28(1.13–9.46)*	

Ref = Reference category; aOR = adjusted Odds Ratio; *=Statistical significance p < 0.05

## Discussion

Immunization has been shown to have a significant impact on vaccine preventable diseases. Despite these success and progress, vaccinations uptake is currently faced with several challenges, such as unequal access, lack of resources and vaccine hesitancy [5, 22–25]. The study found that religion, fathers’ education, and PNC visits are all factors that hinder vaccination uptake in children under one year of age. These findings are similar to other studies that reported culture, religion and community belief systems that were reported as potential barriers or deterrents to vaccine uptake in Africa [22, 26–28]. The study also revealed that children’s vaccination uptake is influenced by the socioeconomic and residential status of their caregivers. Families with higher incomes who live in cities have a better chance of getting their children vaccinated than low-income families who live in rural areas. Similar studies supported the critical influence of family support systems toward improving vaccine coverages among infants in Sub-Saharan African countries [22, 23, 27].

The study found that mothers who visited postnatal care had a higher chance of having their children vaccinated as compared to mothers who had no previous PNC visits. This is consistent with a study conducted in Benin and the findings revealed that children whose mothers had no antenatal care visits had a lower likelihood of receiving full immunization than those whose mothers had 1–3 visits. This demonstrates that contact with health care workers and a better understanding of vaccinations are factors that influence a child being vaccinated. However, institutional mistrust especially across local health authorities within the bigger picture of health systems related factors toward child vaccination uptake has created barriers and unequal access to immunization services and coverages in Africa [22, 24]. Parents can play an important role in ensuring their children are vaccinated, as evidence on the many benefits of vaccinations abound. However, there are other issues relating with the parents that affect vaccine uptake. The study’s findings also revealed that religion played a significant role in children’s vaccination uptake. This is consistent with the study conducted in Nigeria, which shows that religious belief was associated with the non-uptake of vaccination for children by their caregivers[29]. However, this was not the case in Burkina Faso as religion was contributary to partial immunization [30]. Religious beliefs shape how people see the world and determine how they live in many African settings [22, 23]. However, we can argue that individual levels of belief differ across regions and within countries, and thus how people live and what is accepted may influence vaccination uptake [23].

Furthermore, education was identified as a determinant of childhood vaccination uptake in the three countries. The study revealed that fathers with at least a primary level of education have a higher likelihood of their child being vaccinated than those with no formal education. This corroborate findings from a study conducted by Anu Rammohan et al., which revealed that in the six countries studied, having a father with a secondary (high school) education or higher was statistically significant and positively correlated with the likelihood of a child receiving measles vaccination, even if the mother is illiterate[31]. The findings were consistent with the study conducted in southeast Ethiopia that revealed that paternal education was also found to be statistically associated with child immunization status in a cross-sectional study of 591 children aged 12–23 months. In the study, children of fathers with secondary or higher education levels were three times more likely to be fully vaccinated than children whose fathers had no formal education [32]. This could be attributed to cultural influences in various African settings. In many instances, the decision of the family solely depends on the man, reflecting the patriarchal nature of African societies [25, 33]. This is a system where the educational levels of men as heads of households/families continuous to be influenced by culture and tradition.

Awareness of the importance of vaccination, as well as dangers of not being vaccinated demonstrates evidence that may explain why parents who have access to information and watch TV are more likely to have their children fully vaccinated. This is because, they may obtain information from a health facility, the media, or other sources and is consistent with the Ghanaian study [34]. The study findings demonstrate how vaccination knowledge and understanding can significantly influence vaccination uptake among children in African settings. In Mali, studies show that a lack of information and inconvenience led to only partial immunization of children [35]. This means that healthcare workers play a significant role, as do awareness campaigns that could be launched to educate the public and encourage positive behaviour. Using pooled data, from the Gambia, it was found that male children were fully vaccinated more than female children. This significantly differ from results in Liberia and Sierra Leone, where no significant differences were recorded in child sex and vaccination status. Furthermore, the pooled data showed that, the age of parents was significantly higher in Libera when compared to Gambia and Sierra Leone.

The governments of these nations need to strengthen their commitment towards WHO’s 2030 immunization agenda, which envisions a world where everyone, everywhere, at any age, has access to vaccines for good health and well-being [36]. Thus, a value-based Global Immunization Strategy is clearly needed, with the aim of putting citizens/populace at the center [37, 38]. There is also a need to set priorities for action to be implemented in the three African countries studied, for designing of an all-inclusive, integrated, and culturally adaptive new immunization strategies to be implemented. Some unpublished grey literature from Gambia reported that community-based immunization defaulter tracing strategies that are tailored in context-specific local settings have the potential to improve clinic attendance for childhood vaccination. These could also have the potential to influence political decisions that prioritizes immunization programs and strategies for local populace.

### Study limitations

Since this survey was cross-sectional, causal relationships between variables of interest could not be definitively determined. There might be some level of recall bias in the study and non-response could also influence the accuracy of the data.

## Conclusion

The prevalence of childhood vaccination uptake was low among children under 12 months of age and associated factors were number of mothers’ PNC visits, fathers’ educational level, access to watch TV as well as mothers’ number of ANC visits. There is a need to promote the uptake of childhood vaccination uptake across these three countries, especially among rural dwellers. Government should design robust, community-based social and behavioral change communication strategies and programs with strong elements of awareness raising at household and community levels.

## Data Availability

Data for this study were sourced from Demographic Health Survey (DHS)and available here: https://www.dhsprogram.com/data/available-datasets.cfm.
